# Cysteinyl leukotriene receptor 1 modulates autophagic activity in retinal pigment epithelial cells

**DOI:** 10.1038/s41598-020-74755-w

**Published:** 2020-10-19

**Authors:** Andreas Koller, Daniela Bruckner, Ludwig Aigner, Herbert Reitsamer, Andrea Trost

**Affiliations:** 1 Research Program for Experimental Ophthalmology, Department of Ophthalmology and Optometry, University Hospital of the Paracelsus Medical University, Muellner Hauptstrasse 48, 5020 Salzburg, Austria; 2 Institute of Molecular Regenerative Medicine, Spinal Cord Injury and Tissue Regeneration Center, Paracelsus Medical University, Salzburg, Austria

**Keywords:** Autophagy, Macroautophagy, Circadian rhythms, Molecular biology, Transcription, Immunochemistry, Lipid signalling

## Abstract

The retinal pigment epithelium (RPE), which is among the tissues in the body that are exposed to the highest levels of phagocytosis and oxidative stress, is dependent on autophagy function. Impaired autophagy and continuous cellular stress are associated with various disorders, such as dry age-related macular degeneration (AMD), a disease for which effective therapies are lacking. Cysteinyl leukotriene receptor (CysLTR) 1 is a potential modulator of autophagy; thus, the aim of this study was to investigate the role of CysLTR1 in autophagy regulation in the RPE cell line ARPE-19. The polarized ARPE-19 monolayer exhibited expression of CysLTR1, which was colocalized with β-tubulin III. In ARPE-19 cells, autophagic activity was rhythmically regulated and was increased upon CysLTR1 inhibition by Zafirlukast (ZK) treatment. H_2_O_2_ affected the proautophagic regulatory effect of ZK treatment depending on whether it was applied simultaneously with or prior to ZK treatment. Furthermore, mRNA levels of genes related to the leukotriene system, autophagy and the unfolded protein response were positively correlated. As CysLTR1 is involved in autophagy regulation under basal and oxidative stress conditions, a dysfunctional leukotriene system could negatively affect RPE functions. Therefore, CysLTR1 is a potential target for new treatment approaches for neurodegenerative disorders, such as AMD.

## Introduction

Leukotrienes, best known as proinflammatory mediators, are mainly produced by granulocytes, mast cells and macrophages^1^. Arachidonic acid is metabolized by arachidonate 5-lipoxygenase (ALOX5) to leukotriene A_4_ (LTA_4_) and further converted to LTB_4_ and cysteinyl leukotrienes (CysLTs), LTC_4_, LTD_4_ and LTE_4_^1^. The functions of CysLTs, including immune cell recruitment, smooth muscle constriction and promotion of vascular permeability, are mediated via the G-protein-coupled CysLT receptors (CysLTRs) CysLTR1, CysLTR2 and GPR17^1–3^.

In addition to playing immune-related roles, leukotrienes, which regulate noninflammatory activities, are synthesized by diverse nonhematopoietic cells, such as retinal pigment epithelial (RPE) cells^4,5^. It has been postulated that leukotrienes (LTB_4_ and LTC_4_) are involved in the phagocytosis of disks shed by RPE cells in *Xenopus laevis*^4^. Recently, it was reported that inhibition of ALOX5 by PEDF-R peptides increases the survival of RPE cells undergoing oxidative stress^5^. This augmented survival of RPE cells could be explained by the recent finding that LTC_4_ induces an intracellular death-triggering mechanism in the late phase of the unfolded protein response (UPR) caused by excessive endoplasmic reticulum (ER) stress^6^. Upon UPR activation, the membrane-associated CysLTR1 and CysLTR2 are internalized, and CsyLTR1- and CysLTR2-mediated cell death is inhibited by leukotriene receptor antagonists^6^. The UPR induces three distinct pathways via eukaryotic translation initiation factor 2-alpha kinase 3 (EIF2AK3) [PERK], activating transcription factor 6 (ATF6) and inositol-requiring enzyme 1 (IRE1; gene: *ERN1*), leading to activation of the transcription factors ATF4, ATF6 and XBP1, respectively. These three transcription factors have been reported to be important regulators of autophagy under moderate UPR activity^7–9^. Therefore, autophagy is recognized as a cellular prosurvival mechanism^10,11^. As the inhibition of intracellular LTC_4_ receptor signaling keeps the cell in a prosurvival UPR state and moderate UPR activity induces autophagy to cope with ER stress, it seems obvious that leukotriene receptor antagonists could have the potential to regulate autophagy. Recently, Hu et al. demonstrated that blockage of CysLTR1 signaling leads to amelioration of liver injury through activation of autophagy upon aluminum overload^12^.

Autophagy is an intracellular process through which misfolded or long-lived proteins, lipid droplets, invading microorganisms and damaged organelles are degraded and recycled^13^ and an adaptive process that provides nutrients and energy in response to stress, such as starvation or hypoxia. At least three forms of autophagy have been described in mammalian cells: macroautophagy, microautophagy and chaperone-mediated autophagy (CMA)^14^. During microautophagy, cytoplasmic entities destined for degradation are taken up by lysosomes via direct membrane invagination^15^. CMA involves direct delivery of cytosolic proteins targeted for degradation by the constitutive cytosolic chaperone Hsp70 to the lysosomal receptor LAMP-2a, which mediates translocation across the lysosomal membrane^16^. In the present study, we focused on macroautophagy, herein referred to as autophagy. Autophagy is initiated by the formation of a phagophore, which elongates/expands through lipid acquisition from membranes of multiple origins and engulfs a portion of the cytoplasm containing the cargo to be degraded, forming a double membrane autophagosome^17–19^. Autophagosomes fuse with lysosomes to form autolysosomes, and lysosomal proteases catabolize polypeptides to their constituting amino acids, whereas hydrolases degrade lipids, sugars and nucleic acids^20–22^. Therefore, defective autophagic and lysosomal mechanisms can result in accumulation of damaged proteins, leading to cellular degeneration and cell death.

RPE cells form a highly polarized monolayer located between the photoreceptor layer and Bruch’s membrane, the innermost layer of the choroid^23^. RPE cells constitute the outer blood-retina barrier and are essential for nutrition/metabolite circulation between the choroid and the outer retina, as well as for photoreceptor cell metabolism and functioning of the visual cycle^24^. They phagocytose, degrade and recycle photoreceptor outer segments (POSs) for reuse in the visual cycle. In addition to being some of the most active phagocytic cells in the body, these postmitotic cells are constantly exposed to high levels of metabolic and oxidative stress. An age-dependent impairment in the capacity of RPE cells to reduce reactive oxygen species (ROS), leading to further DNA and protein damage and in turn contributing to increased lipofuscin (protein-rich lipid deposits) accumulation, followed by RPE degradation and drusen formation between Bruch´s membrane and the RPE, which are hallmarks of dry age-related macular degeneration (AMD)^25–27^, has been reported^22^. Increases in autophagic proteins (microtubule-associated protein 1 light chain 3 (LC3), ATG7 and ATG9) and the number of autophagosomes, indicating increased autophagic activity, have been reported in the retinas of older individuals compared to those of younger donors. However, in retinas of late AMD patients, the levels of autophagic proteins and number of autophagosomes are lower than those in age-matched controls^28^. Furthermore, impaired autophagic flux has been observed in RPE cells isolated from AMD donors^29^. In agreement with these findings, the autophagy marker Sequestosome-1 (SQSTM) accumulates in AMD patients due to impaired autophagic flux^29,30^. Numerous studies have shown that activation of autophagy increases RPE survival^28,31–33^.

Considering the reduced autophagic activity in RPE cells of AMD patients, agents that activate autophagy may have the ability to improve RPE functions. Thus, the aim of this study was to investigate the role of CysLTR1 in autophagy and UPR regulation in the RPE cell line ARPE-19.

## Results

### CysLTR1 expression in ARPE-19 cells

**Figure 1:**
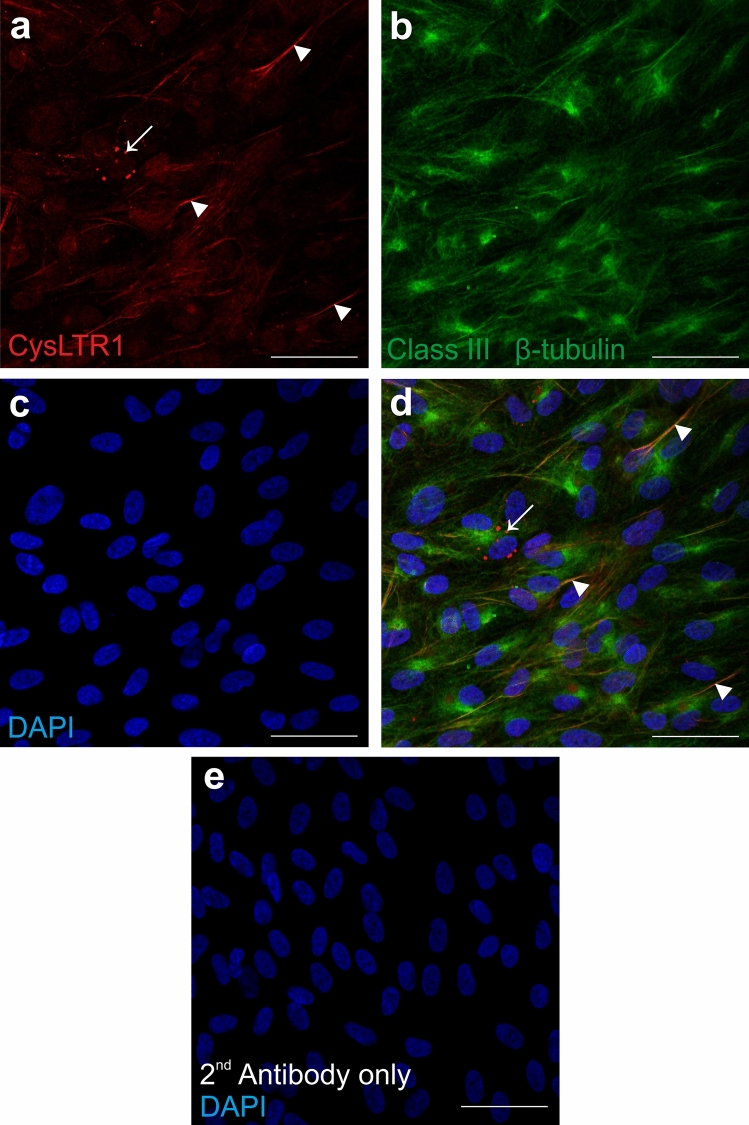
Representative immunofluorescence images of CysLTR1 and Class III β-tubulin in polarized ARPE-19 cells. ARPE-19 cells were cultured for 7 days in low-serum medium. **(a)** CysLTR1 (visualized in red, indicated by white arrowheads), **(b)** class III β-tubulin (green) and **(c)** DAPI (blue) in polarized ARPE-19 cells. **(d)** Merged image of red, green and blue emission showing colocalization of CysLTR1 and class III β-tubulin (yellow, indicated by white arrowheads). Internalized CysLTR1 located near the cell nucleus was observed in individual cells, as indicated by the white arrow. e) Secondary antibody only control + DAPI (blue). Scale bar = 50 µm; n = 5.

As Reynolds et al. already evaluated the protein expression of CysLTR1 in dispersed ARPE-19 cells^34^, the first aim of the present study was to confirm CysLTR1 expression and determine the localization of the receptor in polarized ARPE-19 cells. Immunofluorescence (IF) analysis revealed basolateral CysLTR1 expression in polarized ARPE-19 monolayers (Supplementary Fig. 1) and localization of CysLTR1 to class III β-tubulin-positive cytoskeleton structures (microtubules) (Fig. 1, arrowheads). However, in individual cells of the monolayer CysLTR1 was internalized and located near the nucleus (Fig. 1d, arrow) and was not localized to class III β-tubulin-positive cytoskeleton structures. A specific fluorescence signal was absent in the secondary antibody only control (Fig. 1e).

**Figure 2:**
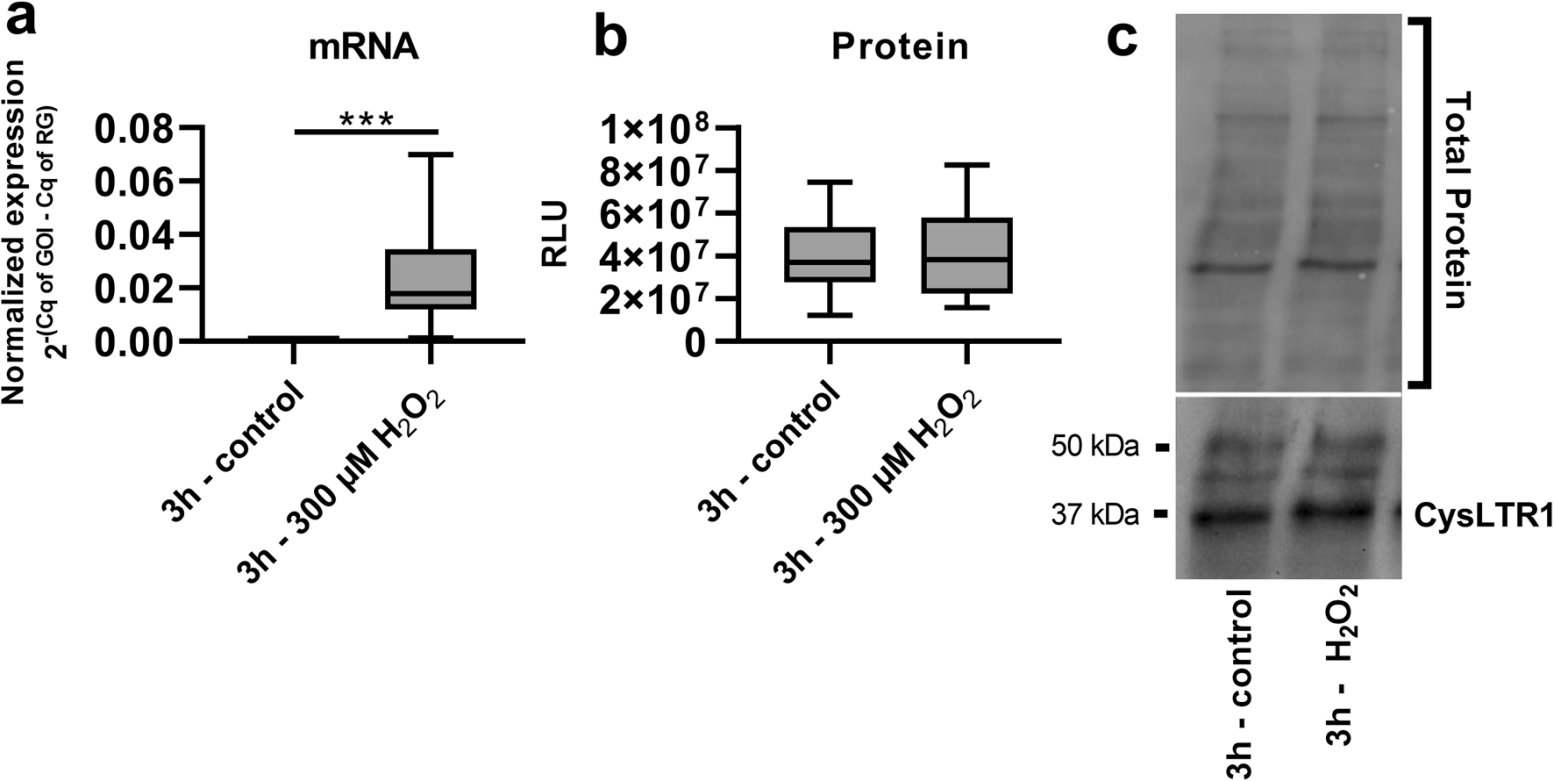
mRNA and protein expression of CysLTR1 in polarized ARPE-19 cells. CysLTR1 expression upon treatment with 300 µM H_2_O_2_ for 3 h in polarized (7–9 days) ARPE-19 cells at the **(a)** mRNA and **(b)** protein levels detected by qPCR and western blot analysis, respectively. **(c)** Representative western blot analysis showing total protein loading and CysLTR1 expression in untreated polarized ARPE-19 cells and polarized ARPE-19 cells treated with H_2_O_2_ for 3 h. The values are represented in box and whisker plot format (min to max); n = 15 (mRNA), n = 12 (protein). Significance was calculated by paired t-test. ***p < 0.001. *Cq* quantification cycle, *GOI* gene of interest, *RG* reference gene.

As ER stress induced by brefeldin A increases CysLTR1 expression^6^, we investigated the effect of H_2_O_2_ as an ER stress inducer on CysLTR1 mRNA and protein expression in polarized ARPE-19 cells. Polarized ARPE-19 cells were left untreated or treated with a nonlethal dose of H_2_O_2_ (300 µM) for 3 h to induce oxidative stress^28,35^. The mRNA expression of CysLTR1 in polarized ARPE-19 cells was very low under basal conditions (p < 0.005, normalized to glucuronidase beta (GUSB)) and was below the detection limit in 9 of 15 samples (Fig. 2a). However, H_2_O_2_ treatment significantly increased CysLTR1 expression levels (mean = 0.023, normalized to GUSB) (Fig. 2a). Western blot analysis confirmed that CysLTR1 protein expression was stable under basal conditions and was not increased upon H_2_O_2_ treatment for 3 h (Fig. 2b,c, Supplementary Fig. 2a,b). The mRNA expression of the two other CysLT receptors, CysLTR_2_ and GPR17, in ARPE-19 cells was not detectable by qPCR (n = 4, data not shown).

### Rhythmic expression of LC3B and UPR transcription factors

**Figure 3:**
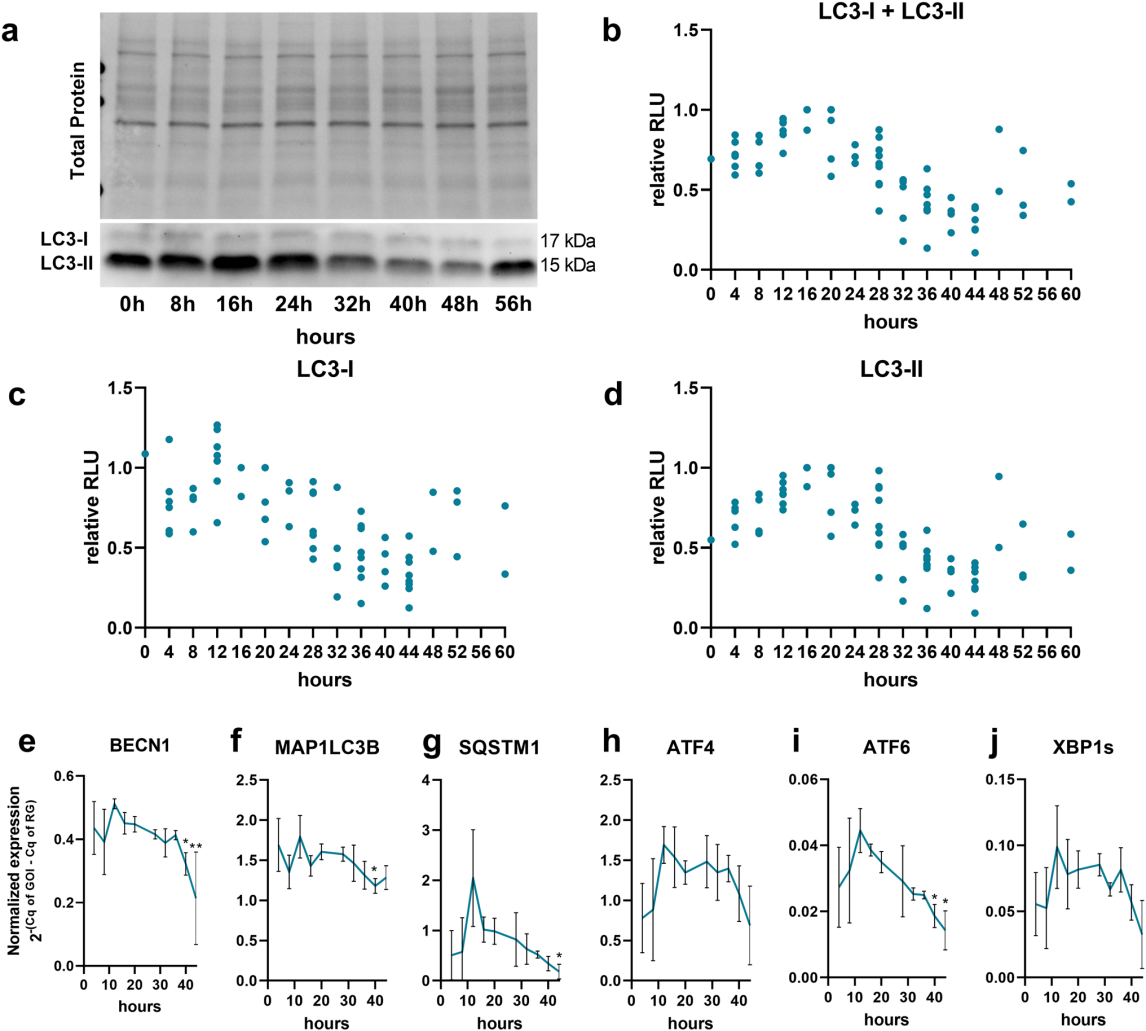
LC3-I and LC3-II protein expression in polarized ARPE-19 cells. **(a)** Representative western blot analysis showing total protein loading and LC3-I and LC3-II expression in polarized ARPE-19 cell samples collected within 52 h. Relative luminescence units (RLUs) of **(b)** LC3-I + LC3-II (representing overall translation of LC3B), **(c)** LC3-I and **(d)** LC3-II in polarized ARPE-19 cell samples collected within a period of 20–52 h. The data were normalized to the amount of total loaded protein and are presented as values relative to those at the time point with the highest LC3-I + LC3-II level in each experiment. The values obtained from individual experiments were combined to visualize rhythmic time-dependent basal autophagic activity (n = 9). Normalized expression of the autophagosomal genes **(e)** BECN1, **(f)** MAP1LC3B, **(g)** and SQSTM1 and the UPR-related transcription factors **(h)** ATF4, **(i)** ATF6, **(j)** and XBP1s in polarized ARPE-19 cells within a period of 40 h. The values are represented as the mean ± standard deviation. Significance was calculated by the Kruskal–Wallis test followed by Dunn’s multiple comparison test. *p < 0.05, **p < 0.01 compared to the time point exhibiting the highest gene expression. *Cq* quantification cycle, *GOI* gene of interest, *RG* reference gene.

Basal autophagic flux in RPE cells exhibits a circadian rhythm in vivo^36^*.* Furthermore, ex vivo cultured ARPE-19 cells express clock and phagocytosis genes in a rhythmic manner^37^. An excellent short summary of the circadian rhythm and clock genes can be found in a review by Cox and Tagahashi^38^. Based on these findings, the potential rhythmic regulation of the autophagic process in polarized ARPE-19 cells was investigated. The LC3-II (lipidated LC3) level is used as a marker of autophagic activity^39,40^. Therefore, LC3-I (unlipidated LC3) and LC3-II levels were analyzed by western blotting (Fig. 3, Supplementary Fig. 2c,d). Cells were not synchronized by serum shock to avoid unknown effects on the intrinsic regulation of basal autophagic activity. Nevertheless, synchronization of three different cell batches was achieved by simultaneous thawing, medium exchange, splitting and polarization. As not all cell batches (n = 9) were synchronized, the data obtained from all time-course experiments of LC3-I and LC3-II expression were combined retrospectively (Fig. 3b). For each time-course experiment, cells were seeded in 12-well plates and expanded until cell confluence was reached. Afterwards, the cells were polarized for 7 days under low-serum conditions. Thus, all generated monolayers used for a single experiment were synchronized (Supplementary Fig. 4). Polarized cells were harvested every 4 or 8 h within a time period of 20–52 h. There was a clear time-dependent alteration of LC3-I and LC3-II protein expression in ARPE-19 cells (Fig. 3a–d). The combined data indicated an autophagic flux period of approximately ≥ 48–56 h (Fig. 3a–d). LC3-I and LC3-II protein levels were regulated concordantly over time (Fig. 3a–d). The expression of the autophagy-related genes BECN1, MAP1LC3B and SQSTM1 at the time points exhibiting the highest and lowest expression levels within 40 h were significantly different (Fig. 3e–g). The expression levels of the UPR transcription factors ATF4, ATF6 and spliced XBP1 (XBP1s), similar to those of autophagic genes, changed within 40 h, but the difference in expression at the time points exhibiting the highest and lowest levels was only significant for ATF6 (Fig. 3h–j). The reference gene GUSB exhibited stable expression over 40 h and was unaffected by rhythmic autophagic activity (Supplementary Fig. 3).

**Figure 4:**
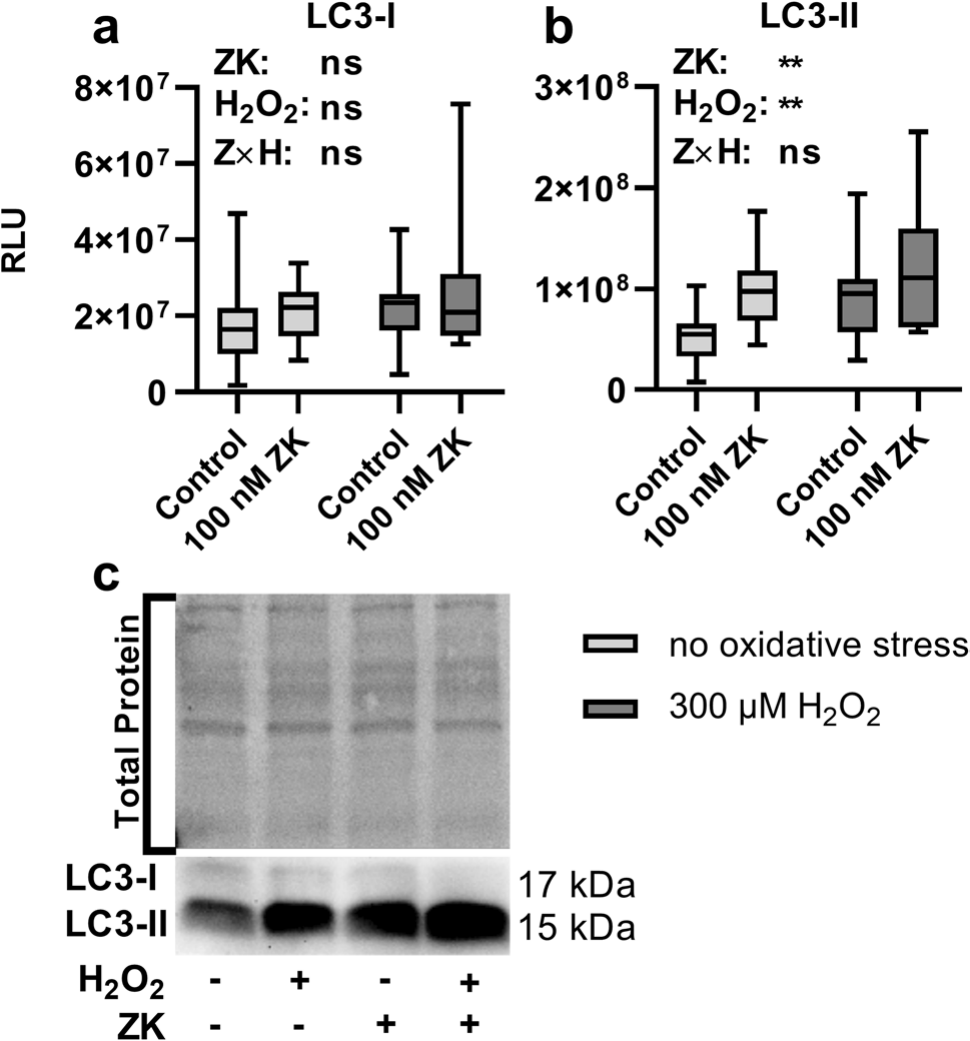
LC3-I and LC3-II protein expression in polarized ARPE-19 cells treated with 100 nM ZK, 300 µM H_2_O_2_ or both 100 nM ZK + 300 µM H_2_O_2_. RLUs of **(a)** LC3-I and **(b)** LC3-II normalized to the amount of total loaded protein in polarized ARPE-19 cells treated with 100 nM ZK, 300 µM H_2_O_2_ or both 100 nM ZK + 300 µM H_2_O_2_ for 3 h. **(c)** Representative western blot analysis showing total protein loading and LC3-I and LC3-II expression in polarized ARPE-19 cells treated with ZK and H_2_O_2_. ARPE-19 cells were cotreated with 10 µg/ml lysosomal degradation inhibitors E64d and pepstatin A to prevent LC3-II degradation. The values are represented in box and whisker plot format (min to max); n = 14. The significance of differences in LC3-I and LC3-II expression upon ZK, H_2_O_2_ and ZK + H_2_O_2_ treatment was calculated by repeated measures two-way ANOVA (main factors: ZK treatment (matched) and H_2_O_2_ treatment (matched); interaction: ZK × H_2_O_2_). *p < 0.05, **p < 0.01.

### Increased autophagic activity upon leukotriene receptor inhibition

As polarized ARPE-19 cells express the CysLTR1 protein and because H_2_O_2_, a known autophagy modulator^28^, increases CysLTR1 mRNA levels, cells were left untreated (3 h) or treated with 100 nM CysLTR1 receptor antagonist zafirlukast (ZK), a nonlethal dose of H_2_O_2_ (300 µM) or a combination of both for 3 h. Rhythmic regulation of LC3-I and LC3-II (see Fig. 3) was not considered for cell treatments; nevertheless, the cells exposed to each treatment were compared to a time-matched control sample (all generated monolayers used for a single experiment were synchronized, Supplementary Fig. 4). To prevent LC3-II degradation, the cells were cotreated with 10 µg/ml E64d and 10 µg/ml pepstatin A. Increased levels of lipidated LC3-II in the presence of lysosomal degradation inhibitors indicate amplified autophagic flux^39,40^. LC3-I and LC3-II protein expression levels were analyzed by western blotting (Fig. 4, Supplementary Fig. 6). H_2_O_2_ treatment of ARPE-19 cells did not affect LC3-I levels, but LC3-II levels were significantly increased in ARPE-19 cells (Fig. 4a–c) compared to the untreated control sample. Compared to the untreated controls, ZK treatment significantly increased LC3-II levels in unchallenged (no oxidative stress) and 300 µM H_2_O_2_-treated cells (Fig. 4b,c) but not on LC3-I levels (Fig. 4a, c). The optimal ZK concentration for cell treatment was determined by a small preliminary experiment, it was found that 100 nM ZK effectively activated autophagic activity through CysLTR1 inhibition (Supplementary Fig. 5). Additionally, the effect of CysLTR1 inhibition by ZK (100 nM) treatment on caspase 3/7 activity in untreated and 300 µM H_2_O_2_-treated ARPE-19 cells was investigated after a period of 3, 6, 24 or 48 h. Interestingly, caspase 3/7 activity in polarized ARPE-19 cells was significantly increased after 24 and 48 h under basal and oxidative stress (300 µM H_2_O_2_) conditions. CysLTR1 inhibition led to an overall significant reduction in caspase 3/7 activity under basal cell conditions but not in cells exposed to oxidative stress (300 µM H_2_O_2_) (Supplementary Fig. 7a,b).

**Figure 5:**
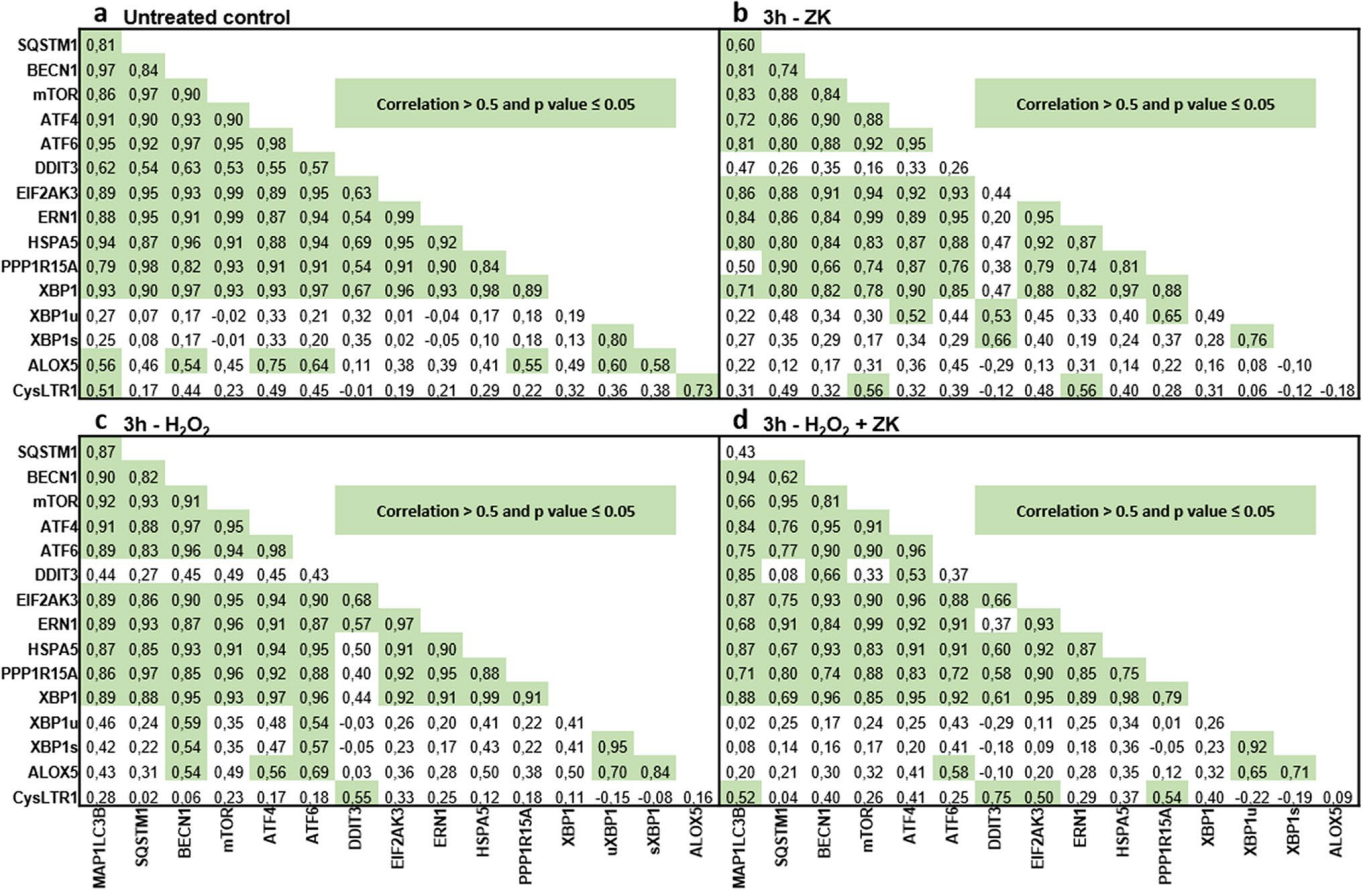
Pearson R correlation matrix of autophagosomal (MAP1LC3B, SQSTM1, BECN1 and mTOR), UPR-related (ATF4, ATF6, DDIT3, EIF2AK3, ERN1, HSPA5, PPP1R15A, XBP1 [total XBP1], XBP1u [unspliced], and XBP1s [spliced]), and leukotriene synthesis (ALOX5) genes and CysLTR1 in **(a)** untreated control polarized ARPE-19 cells and polarized ARPE-19 cells treated with **(b)** 100 nM ZK, **(c)** 300 µM H_2_O_2_ and **(d)** 300 µM H_2_O_2_ + 100 nM ZK for 3 h (n = 16).

### Correlation of autophagic, UPR-related and leukotriene-related gene expression

As LC3-I and LC3-II proteins, autophagic genes and UPR transcription factor genes are expressed in a rhythmic manner (Fig. 3)^36,41,42^, the potential correlations between autophagic, UPR-related and leukotriene system-related genes were investigated. Overall, the expression levels of autophagic and UPR-related genes were significantly positively correlated (Fig. 5a). Furthermore, ALOX5 expression was highly correlated with the expression of autophagy-related genes (MAP1LC3B and BECN1), UPR-related genes (ATF4, ATF6, XBP1s, unspliced XBP1 [XBP1u] and PPP1R15A [GADD34]) and CysLTR1 (Fig. 5a). CysLTR1 expression was further positively correlated with MAP1LC3B expression (Fig. 5a). Treatment of ARPE-19 cells with 100 nM ZK for 3 h altered specific gene correlations, especially correlations with the expression of the UPR-related gene DDIT3 (CHOP), and resulted in a loss of previously observed correlations between the expression of ALOX5 and the expression of MAP1LC3B, BECN1, ATF4, ATF6, XBP1s, XBP1u, PPP1R15A and CysLTR1 (Fig. 5b). However, upon ZK treatment, CysLTR1 expression was significantly correlated with mTOR and ERN1 (IRE1α) expression (Fig. 5b). Furthermore, H_2_O_2_ treatment (300 µM) affected the correlations of gene expression, including correlations between the expression of DDIT3, XBP1u, XBP1s, ALOX5, and CysLTR1 and especially correlations between CysLTR1 expression and the expression of ALOX5 and DDIT3 (Fig. 5c). Cotreatment with H_2_O_2_ and ZK in turn averted the loss of correlation between DDIT3 expression and the expression of various autophagic and UPR-related genes and abolished the strong correlations between BECN1 expression and the expression of XBP1u and XBP1s (Fig. 5d).

**Figure 6:**
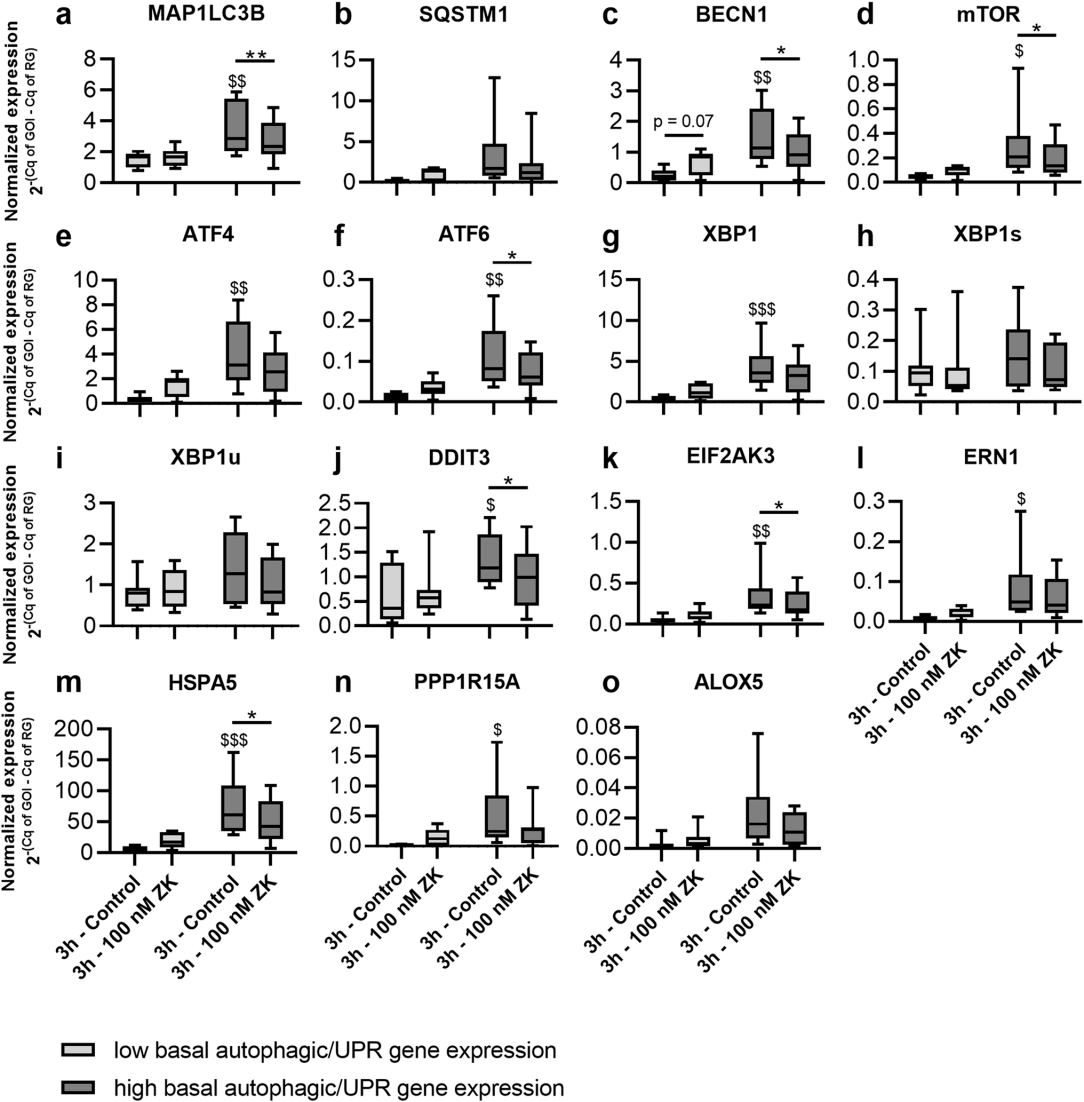
Normalized expression of the autophagosomal **(a)** MAP1LC3B, **(b)** SQSTM1, **(c)** BECN1 and **(d)** mTOR, UPR-related, **(e)** ATF4, **(f)** ATF6, **(g)** XBP1, **(h)** XBP1s, **(i)** XBP1u, **(j)** DDIT3, **(k)** EIF2AK3, **(l)** ERN1, **(m)** HSPA5, **(n)** PPP1R15A and leukotriene synthesis **(o)** ALOX5 genes in polarized ARPE-19 cells treated with 100 nM ZK for 3 h compared to time-matched untreated controls. ARPE-19 cells were separated into low (n = 7) and high (n = 8) autophagy-/UPR-related gene-expressing cells. The values are represented in box and whisker plot format (min to max). The significance of differences in gene regulation upon classification and ZK treatment were calculated by repeated measures two-way ANOVA (main factors: low & high autophagy-/UPR-related gene-expression and ZK treatment (matched); interaction: autophagy-/UPR-related gene-expression × ZK) followed by Bonferroni’s multiple comparison test. *p < 0.05, **p < 0.01 as indicated; $p < 0.05, $$ < 0.01, $$ < 0.001 compared to low autophagy-/UPR-related gene-expressing cells. *Cq* quantification cycle, *GOI* gene of interest, *RG* reference gene.

In addition to analyzing correlations in the gene expression data, we compared expression data from untreated and time-matched 100 nM ZK-treated polarized ARPE-19 cells. Concordant with the autophagic activity rhythm observed in polarized ARPE-19 cells, as shown in Fig. 3 (e–j, LC3-I and LC-II protein), autophagic and UPR-related genes also exhibited dynamic expression within the 40-h rhythmic period analyzed. Thus, the time point of treatment within the rhythm affected the impact of CysLTR1 inhibition on autophagy- and UPR-related mRNA expression. Therefore, the untreated control samples were subsequently separated into two groups: those expressing low levels of autophagy-/UPR-related genes and those expressing high levels of autophagy-/UPR-related genes (the median of the mean expression levels of BECN1, MAP1LC3B, SQSTM1, mTOR, ATF4, ATF6 and XBP1 normalized to GUSB was used for group classification; < median = low basal autophagy-/UPR-related gene expression, ≥ median = high basal autophagy-/UPR-related gene expression). Thus, the two groups roughly represented two phases within the autophagic rhythm. The expression of the autophagy-related (MAP1LC3B, BECN1, and mTOR) and UPR-related (ATF4, ATF6, XBP1 DDIT3, EIF2AK3, ERN1, HSPA5 and PPP1R15A) genes differed significantly between low and high autophagy-/UPR-related gene-expressing groups (Fig. 6a,c–g,j–n). Treatment of polarized ARPE-19 cells with 100 nM ZK for 3 h showed a trend (p = 0.07) to increase BECN1 expression in low autophagy-/UPR-related gene-expressing cells (Fig. 6c) but significantly decreased BECN1, MAP1LC3B, mTOR, ATF6, DDIT3, EIF2AK3 and HSPA5 expression (Fig. 6a,c,d,f,j,k,m) in high autophagy-/UPR-related gene-expressing cells. Expression levels of SQSTM1 and ALOX5 were not significantly affected by 100 nM ZK treatment (3 h) (Fig. 6b,o). Furthermore, the impact of CysLTR1 inhibition on autophagy- and UPR-related genes in ARPE-19 cells subjected to oxidative stress (300 µM H_2_O_2_) was investigated. Compared to H_2_O_2_ treatment alone, cotreatment with 300 µM H_2_O_2_ and 100 nM ZK for 3 h significantly increased XBP1u expression and decreased CysLTR1 expression in polarized ARPE-19 cells (Supplementary Fig. 8 i,p).

**Figure 7:**
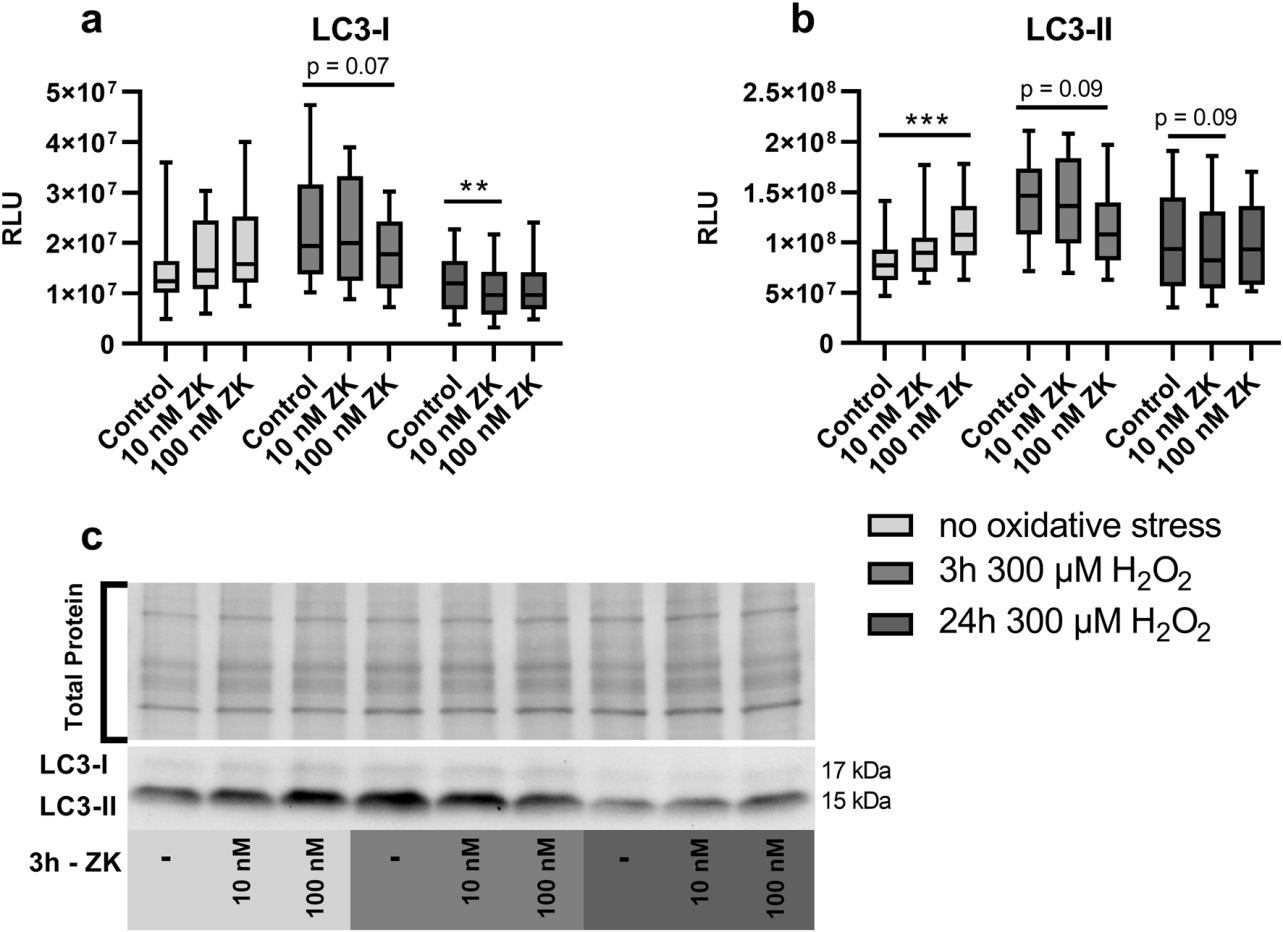
Effect of ZK treatment on LC3-I and LC3-II protein expression in polarized ARPE-19 cells pretreated with H_2_O_2_. RLUs of **(a)** LC3-I and **(b)** LC3-II normalized to the amount of total loaded protein in polarized ARPE-19 cells prior to H_2_O_2_ exposure and polarized ARPE-19 cells challenged with 300 µM H_2_O_2_ for 3 h or 24 h followed by a resting period (control) or treatment with 10–100 nM ZK for 3 h. **(c)** Representative western blot analysis showing total protein loading and LC3-I and LC3-II in polarized ARPE-19 cells prior H_2_O_2_ exposure and polarized ARPE-19 cells challenged with H_2_O_2_ for 3 h or 24 h followed by ZK treatment for 3 h. ARPE-19 cells were cotreated with 10 µg/ml E64d and pepstatin A (lysosomal degradation inhibitors) to prevent LC3-II degradation upon H_2_O_2_ challenge. The data are presented in box and whisker plot format (min to max); n = 10. Significance was calculated by repeated measures two-way ANOVA (main factors: H_2_O_2_ exposure (matched) and ZK treatment (matched); interaction H_2_O_2_ exposure × ZK) followed by Bonferroni’s multiple comparison test. **p < 0.01, ***p < 0.001.

As H_2_O_2_ treatment induced CysLTR1 gene expression and CysLTR1 inhibition during challenge with H_2_O_2_ for 3 h further increased autophagic flux, the impact of CysLTR1 inhibition on autophagic activity following short and long periods of H_2_O_2_ exposure was determined. Polarized ARPE-19 cells were challenged with 300 µM H_2_O_2_ for 3 or 24 h. Afterwards, the cells were incubated in the absence of H_2_O_2_ for an additional 3 h without or with 10 or 100 nM ZK. ZK treatment without prior H_2_O_2_ challenge served as a control “no oxidative stress” condition. ZK (100 nM) led to a significant increase in LC3-II but not LC3-I in unchallenged ARPE-19 cells compared to untreated control samples (Fig. 7a–c, Supplementary Fig. 9). The lower concentration of ZK (10 nM) had no significant effect on LC3-I and LC3-II expression (Fig. 7a–c). Interestingly, compared to control treatment or treatment with 300 µM H_2_O_2_ for 3 h followed by culture in fresh medium without treatment for 3 h, treatment with 100 nM ZK for 3 h following a short period of H_2_O_2_ challenge (3 h) induced a trend toward reduced LC3-I and LC3-II expression (Fig. 7a–c). The lower ZK concentration (10 nM) had no effect on LC3-I and LC3-II expression following a short period of H_2_O_2_ exposure (Fig. 7a–c). Compared to control treatment with 300 µM H_2_O_2_ for 24 h followed by culture in fresh medium without treatment for 3 h, CysLTR1 inhibition by 10 nM ZK but not 100 nM ZK led to a significant decrease in LC3-I levels and a trend toward reduced LC3-II levels (Fig. 7a–c).

## Discussion

Autophagy dysfunction and cellular stress increase with age and are associated with various diseases, including neurodegenerative disorders^43,44^. RPE cells are exposed to high levels of phagocytosis and oxidative stress; hence, functional autophagy is essential for ensuring the integrity of the RPE^22^. Accordingly, impaired autophagy in RPE cells is a main characteristic of dry AMD, the most common cause of blindness in individuals over 60 years of age in Western countries^26,27^. Thus, the interplay between ER stress and autophagy is a highly relevant topic of current research^45^. CysLTR1 has the potential to be an essential modulator of the autophagic process^12^ and to play a crucial role during ER stress^6^; therefore, we hypothesize that there is a strong association between CysLTR1 activity, ER stress and autophagy in RPE cells.

ARPE-19 cells are able to generate polarized monolayers to mimic a functional RPE in vitro^46^. It should be noted that ARPE-19 cells are of male origin and that the *CYSLTR1* gene is located on the X chromosome; thus, a sex difference cannot be excluded and should be considered in future experiments using primary RPE cells. Although rather low CysLTR1 mRNA levels were found under basal conditions, stable protein expression was observed in ARPE-19 cells by IF and western blot analysis. Interestingly, CysLTR1 was located basolaterally in the polarized monolayer in vitro and was localized to the microtubules of the cells. In vivo, the microtubules of RPE cells are involved in POS phagocytosis and are important for the intracellular transport of (auto)phagosomes^47–49^. A correlation between leukotriene activity in RPE cells of *Xenopus laevis* and POS phagocytosis was already postulated in 1989 by Birkle et al.^4^. Taken together, these data indicate the involvement of CysLTR1 in phagocytic processes or phagosome transport. In individual cells CysLTR1 was localized intracellularly near the nucleus, as reported by Dvash et al.^6^. Whether the membrane-bound or intracellular localization of CysLTR1 indicates that the receptor plays a role in distinct mechanisms or has a functional dependency should be clarified in future studies. ER/oxidative stress induction by H_2_O_2_ treatment for a short period, which also affects autophagic activity^44^, increased CysLTR1 expression at the mRNA level; however, the amount of CysLTR1 protein did not differ between untreated and H_2_O_2_-treated (3 h) cells. Receptor mRNA transcription, protein translation, localization and degradation are very dynamic processes that permanently affect mRNA/protein levels and may explain the observed discrepancy between mRNA and protein levels following H_2_O_2_ treatment. Nevertheless, the induction of CysLTR1 mRNA upon H_2_O_2_ treatment highlights the potential importance of the leukotriene system in ER/oxidative stress and autophagy.

Numerous cellular functions are regulated by a daily rhythm, known as the circadian rhythm. Hence, the mammalian retina contains a circadian clock system, and following POS shedding, the phagocytosis and recycling of POSs by RPE cells is time-dependent^50^. Thus, basal autophagy in murine RPE cells is rhythmically regulated, as visualized by peaks of LC3-II levels and the number of autophagosomes over a period of time^36^. Interestingly, ARPE-19 cells exhibit rhythmic expression of clock and phagocytosis genes in vitro^37^. In line with this previous study, rhythmic regulation of autophagic activity in the absence of external stimuli was observed in the present study. Furthermore, autophagic genes and UPR transcription factors are expressed in a comparable rhythm. It was previously reported that basal UPR activity in the mouse liver is cyclic^42^. The interplay between autophagy and UPR activity is the focus of research, especially research on neurodegenerative diseases^51^. We observed positive correlations between almost all investigated autophagy- and UPR-related genes, which further highlights a fundamental dependency of these two systems in RPE cells. Milicevic et al. reported rhythmic expression of clock and phagocytosis genes lasting < 24 h, which is different from the rhythm of ≥ 48–56 h observed in the present study. This discrepancy may be explained by the serum shock used to synchronize the cell rhythm applied by Milicevic et al.^37^. Nevertheless, ARPE-19 cells follow an intrinsic rhythmic cell metabolism that is independent of external stimuli. Thus, dynamic autophagic activity should be considered when working with ARPE-19 cells in vitro. Whether the membrane-bound or intracellular localization of CysLTR1 is associated with the rhythmic regulation of phagocytosis, phagosome transport, autophagy or basal UPR activity is of interest for future studies. In particular, as basal mRNA levels of ALOX5 and CyLTR1 are positively correlated with rhythmically regulated expression of autophagy- and UPR-related genes, this correlation indicates a time-dependent activity of the leukotriene system in human RPE cells.

Although different batches of ARPE-19 at different passage numbers (P9-15) were treated at different time points within the autophagic rhythm in the present study, LC3-II protein levels were consistently higher in ZK-treated cells than in time-matched controls. An increase in LC3-II levels in the presence of lysosomal degradation inhibitors is indicative of increased autophagic flux^39,40^. Therefore, the increase in LC3-II protein levels following ZK treatment in the current experiments indicates that CysLTR1 is involved in autophagic flux regulation. However, due to variations in the gene expression of autophagy- and UPR-related genes during the intrinsic autophagic rhythm, two groups roughly representing two phases within the autophagic rhythm were defined based on the expression of these genes and analyzed. This biphasic analysis revealed differences in gene expression following inhibition of CysLTR1 signaling. In cells expressing low levels of autophagy-/UPR-related genes, ZK treatment did not significantly affect the expression of autophagic genes. In contrast, CysLTR1 inhibition in cells expressing high levels of autophagy-/UPR-related genes significantly reduced the expression of various autophagy-/UPR-related genes. This reduction could be a consequence of a negative feedback loop, as CysLTR1 was inhibited in the presence of high levels of basal autophagy and UPR-related gene expression^52,53^. Overall, CysLTR1 inhibition modulated the expression of autophagy- and UPR-related genes and completely abolished the correlation between ALOX5 expression and the aforementioned autophagy-/UPR-related gene expression, suggesting self-regulation of CysLTR1 signaling. The rhythm-independent regulation of LC3-II and the rhythm-dependent modulation of gene expression by CysLTR1 inhibition could be explained by a direct effect on autophagosome formation and additional modulation of transcriptional processes. Recently, PERK and ATF4 were reported to modulate autophagy in two distinct ways, namely, at the transcriptional level and through direct regulation of autophagosome formation at the protein level^7^. The combination of these data with our findings suggests a potential interplay between CysLTR1 signaling and the PERK-ATF4 axis that represents a promising target for future studies.

In the present study, H_2_O_2_ was used to mimic cellular oxidative stress^28,35,54,55^. Cotreatment with H_2_O_2_ and ZK resulted in increased autophagic activity; however, treatment of ARPE-19 cells with ZK following prior exposure to H_2_O_2_ did not increase autophagy but induced a trend toward reduced autophagic activity. CysLTR1 inhibition significantly reduced caspase 3/7 activity in unstressed cells, which highlights the relationship between CysLTR1 activity and apoptosis under basal cell conditions. Interestingly, under oxidative stress, CysLTR1 inhibition had no effect on caspase 3/7 activity. These data likely indicate that CysLTR1 signaling is endogenously regulated upon/during an oxidative stress response and that CysLTR1 plays a dual role under basal and oxidative stress conditions, which should be investigated in future studies.

In summary, ARPE-19 cells exhibited rhythmic basal autophagic activity in the absence of external stimuli. Furthermore, CysLTR1 was mostly expressed basolaterally in RPE cells, and inhibition of CysLTR1 resulted in an increase in autophagic activity. The expression of leukotriene-related genes was correlated with that of autophagy- and UPR-related genes under basal and cellular stress conditions, and oxidative stress drastically increased CysLTR1 mRNA levels and altered the potential of CysLTR1 inhibition to regulate autophagic activity.

CysLTR1 inhibition by ZK or Montelukast has been used to treat asthma since the nineties^3^, but the potential of CysLTR1 as a pharmacological target for the treatment of neurodegenerative disorders, such as Alzheimer’s disease (AD), has become a topic of interest because the leukotriene system targets AD pathologies on multiple levels^56^. Similarly, dry AMD is a multifactorial neurodegenerative disease with limited treatment possibilities^57^. Most interestingly, the leukotriene system was recently described as a potential target for therapeutic approaches for the exudative form of late AMD (wet AMD), as diverse leukotriene inhibitors reduce choroidal neovascularization in murine wet AMD models^58^. Because inhibition of the leukotriene system also induces autophagy, inhibits cell death and exerts immunomodulatory effects, it represents a potential pharmaceutical target for the treatment of not only wet but also dry AMD^1,5,6,12,58^. Therefore, gaining a fundamental understanding of physiological and pathophysiological leukotriene-dependent effects in AMD-associated cellular systems seems to be a promising strategy for the development of new therapeutic strategies.

## Methods

### Cell lines

The human RPE cell line ARPE-19 (male origin, obtained from ATCC, VA, USA) was cultivated in DMEM:F12 (ATCC) containing 10% fetal bovine serum (FBS, Thermo Fisher Scientific, MA, USA). For all experiments, the cells were expanded in medium containing 10% FBS until 100% cell confluence was reached. Afterwards, ARPE-19 cells were polarized for 7–9 days in DMEM:F12 with 2% FBS to generate cell monolayers. Cell cultivation and experiments were performed under standardized normoxic conditions in a 37 °C humidified incubator with 5% CO_2_.

### Cell treatment

Polarized ARPE-19 cells were left untreated or treated with 10, 100 or 1000 nM ZK (Selleckchem, TX, USA) for 3, 6, 24 or 48 h (DMSO concentration ≤ 0.001%). For 48-h treatments, the compound-containing medium was renewed after 24 h. To prevent LC3-II degradation for protein analysis, the cells were cotreated with 10 µg/ml E64d (Selleckchem) and 10 µg/ml pepstatin A (Selleckchem) (DMSO concentration ≤ 0.2%). All treated cell samples were compared to a time-matched untreated control sample.

### RNA isolation/cDNA synthesis/qPCR

Polarized ARPE-19 monolayers were generated and treated in 12-well plates (Sigma-Aldrich, MO, USA). mRNA was isolated using the High Pure RNA Isolation Kit (Roche, Switzerland), and then cDNA was synthesized (iScript Kit, Bio-Rad, CA, USA) according to the manufacturer’s protocols. Quantitative PCR (qPCR) was performed with BRYT Green dye-based GoTaq qPCR Master Mix (Promega, WI, USA) on the CFX96 system (Bio-Rad) using the specific primers listed in Table 1. Expression data were normalized to the level of the reference gene GUSB using CFX Manager Software (Bio-Rad). For the comparison of untreated and 100 nM ZK-treated cells, the expression data for untreated cells (= control) were separated into two groups of low and high autophagy-/UPR-related gene expression roughly representing roughly two phases of the autophagic rhythm based on the expression of a combination of seven genes (see below). The median of the mean expression of BECN1, MAP1LC3B, SQSTM1, mTOR, ATF4, ATF6 and XBP1 normalized to the expression of GUSB was used for group classification (< median = low basal autophagy-/UPR-related gene expression, ≥ median = high basal autophagy-/UPR-related gene expression).

**Table 1:** Primer sequences for qPCR.

	Forward 5′–3’	Reverse 5′–3’
GUSB	AGCGAGTATGGAGCAGAAAC	TGATCCAGACCCAGATGGTA
MAP1LC3B	CGTCGGAGAAGACCTTCAAG	CTGCTTCTCACCCTTGTATCG
SQSTM1	TGAAACACGGACACTTCGG	TCAGGAAATTCACACTCGGATC
BECN1	ACGAGTGTCAGAACTACAAACG	TTTCCACATCTTCCAGCTCC
mTOR	TTCGTGCCTGTCTGATTCTC	ATCCCGATTCATGCCCTTC
ATF4	ATGGGTTCTCCAGCGACAAG	GGCATCCAAGTCGAACTCCT
ATF6	GCCGCCGTCCCAGATATTA	CCGAGTTCAGCAAAGAGAGC
DDIT3	GTTAAAGATGAGCGGGTGGC	GCTTTCAGGTGTGGTGATGTATG
EIF2AK3	ACGATGAGACAGAGTTGCGA	TGCTAAGGCTGGATGACACC
ERN1	CGGCCTCGGGATTTTTGGAA	TGCCATCATTAGGATCTGGGAG
HSPA5	TTGGAGGTGGGCAAACAAAG	GTCTTTGGTTGCTTGGCGTT
PPP1R15A	GACTGCAAAGGCGGCTCAAG	AGACAGCCAGGAAATGGACAG
XBP1	GCGCTGAGGAGGAAACTGAAA	AAGGCCATGAGTTTTCTCTCGT
XBP1u	CAGACTACGTGCACCTCTGC	CTGGGTCCAAGTTGTCCAGAAT
XBP1s	GCTGAGTCCGCAGCAGGT	CTGGGTCCAAGTTGTCCAGAAT
ALOX5	CATCGAGTTCCCCTGCTACC	ACGTCGGTGTTGCTTGAGAA
CysLTR1	TCATAACCTTGTCTCTGGCTG	CTGGGTACATAAGTCACGCTG
CysLTR2	AAGGCAGGAGAGATAGAGGCA	TTGAGGAACTCTCCATACCTTGC
GPR17	CCCCAAGAGAGATGCTGAAAC	CAGGGAGAAGTTGGTGATCAG
SDHA	TTGTTCAGTTCCACCCTACAG	GGGCGTATCGCTCCATAAA
IPO8	CTATGTGGAGATGCAGGAGAAG	CAAGTTGAACGAAGAGTGGAATG

### Western blot analysis

Polarized ARPE-19 monolayers were generated and treated in 12-well plates. Proteins were isolated with RIPA lysis buffer (Santa Cruz Biotechnology, USA), separated by SDS page using 10% (receptor analysis) or AnykD (LC3B analysis) TGX stain-free gels (Bio-Rad) and transferred to a PVDF membrane (Amersham Hybond, GE Healthcare, IL, USA) by wet electroblotting (Bio-Rad). Protein expression was normalized to the amount of total loaded protein (Image Lab 6.0.1, Bio-Rad). For detection of CysLTR1, the membrane was blocked with EveryBlot (Bio-Rad) for 10 min at room temperature and incubated overnight with an anti-CysLT1 antibody (ab151484, Abcam) diluted 1:500 in EveryBlot blocking solution at room temperature. CysLTR1 was visualized using an anti-rabbit antibody conjugated to HRP (Agilent, CA, USA) and Clarity Western ECL Substrate (Bio-Rad). For detection of LC3-I and LC3-II, the membrane was blocked with 5% milk powder for 1 h at room temperature and incubated overnight with a recombinant anti-LC3B antibody [EPR18709] (ab192890, Abcam, UK) diluted 1:1000 in blocking solution at 4 °C. LC3B was visualized using an anti-rabbit antibody conjugated to HRP and Clarity Western ECL Substrate. Chemiluminescence was detected using the ChemiDoc XRS + system (Bio-Rad). Full-length blots are presented in the supplementary material.

### IF microscopy

Polarized ARPE-19 monolayers were generated in 8-well chamber slides (Corning, NY, USA). Antibody-based IF was performed on RPE monolayers generated from ARPE-19 cells as described previously^59^. CysLTR1 was visualized using an anti-CysLT1 antibody (1:100, ab151484, Abcam) and a donkey anti-rabbit antibody conjugated to Alexa Fluor 555 (1:1000, A-31572, Thermo Fisher Scientific). β-Tubulin III was visualized using an anti-β III tubulin antibody (1:200, G7121, Promega) and a donkey anti-mouse antibody conjugated to Alexa Fluor 488 (1:1000, A-21202, Thermo Fisher Scientific). For secondary antibody only controls, primary antibodies were omitted during incubation, and the samples were only exposed to fluorescence-labeled secondary antibodies.

### Documentation

IF images were taken by using a confocal laser-scanning unit (Axio Observer Z1 attached to LSM710, Zeiss, Germany;  40 × oil immersion objective lens, numerical aperture 1.30, Zeiss). The single optical section mode of the confocal microscope was used for image acquisition to document pixel colocalization of different channels with appropriate filter settings for Alexa Fluor 488 (495 nm excitation), Alexa Fluor 555 (555 nm excitation) and DAPI (345 nm excitation).

### Caspase 3/7 activity assay

Polarized ARPE-19 monolayers were generated and treated in white 96-well microplates (Thermo Fisher Scientific). The Caspase-Glo 3/7 Assay (Promega) was performed according to the manufacturer’s protocol. Luminescence was detected using a DTX880 Multimode Detector (Beckman Coulter, CA, USA).

### Statistical analysis

Statistical analysis was performed using GraphPad Prism 8.2.0 (GraphPad Software, Inc., CA, USA), and the analysis methods are described in each figure legend.

## Supplementary information

Supplementary Information.
