# Supplementary material for: Cysteinyl leukotriene receptor 1 modulates autophagic activity in retinal pigment epithelial cells
Source: Sci Rep. 2020 Oct 19;10:17659. doi: 10.1038/s41598-020-74755-w (PMC7573618; doi:10.1038/s41598-020-74755-w)
Supplement: Supplementary file 1 — Supplementary Information. [file 41598_2020_74755_MOESM1_ESM.pdf]

Supplementary Information

Cysteinyl leukotriene receptor 1 modulates autophagic activity in retinal pigment epithelial cells

Andreas Koller, Daniela Bruckner, Ludwig Aigner, Herbert Reitsamer and Andrea Trost

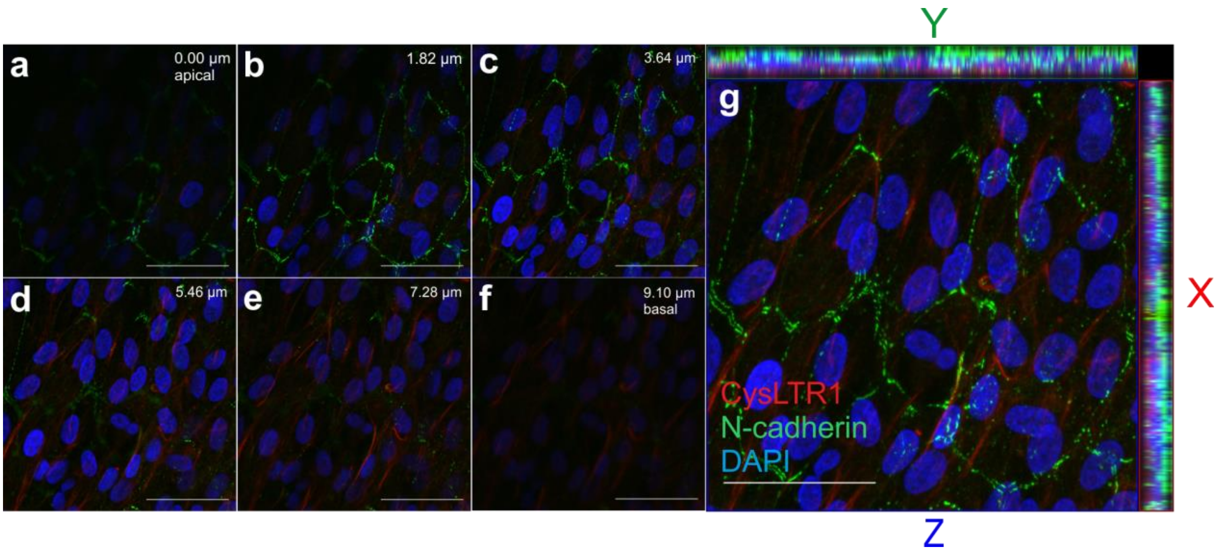

Supplementary Figure 1: Z-stack images of CysLTR1 (red), N-cadherin (1:100, N-cadherin (D-4), sc-8424, Santa Cruz; green) and DAPI (blue) in polarized ARPE-19 cells. Images were collected at 1.82 µm sections starting at a) 0.00 µm, b) 1.82 µm, c) 3.64 µm, d) 5.46 µm, e) 7.28 µm and f) 9.10 µm (apical – basal). g) The maximum intensity projection of all sections (capital letter X, Y and Z are the cut lines). CysLTR1 is expressed mainly basolateral in the ARPE-19 cell monolayer. Scale bar = 50 µm.

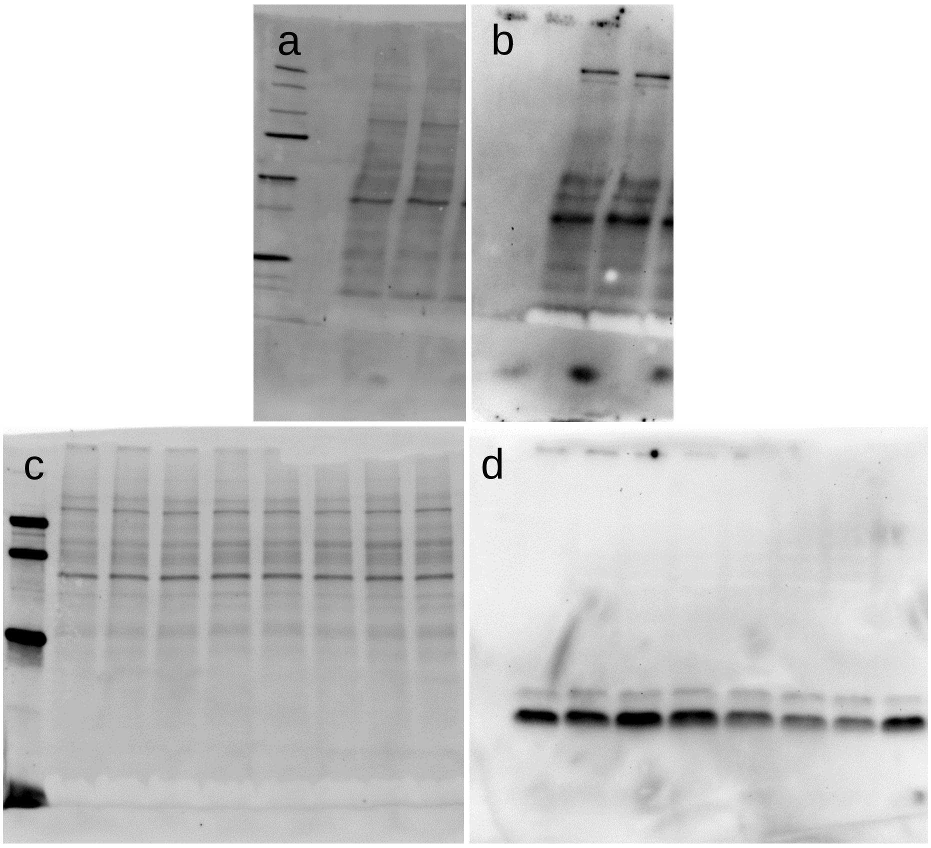

Supplementary Figure 2: Full length blots of figure 2 and 3. a, c) Total protein loading detected after blotting using stain-free imaging technology (Bio-Rad). b) Detection of CysLTR1 using Anti-CysLT1 antibody (ab151484, Abcam). d) Detection of LC3-I and LC3-II protein using recombinant Anti-LC3B antibody [EPR18709] (ab192890, Abcam). Marker: Precision Plus Protein Unstained Standards (a), Precision Plus Protein WesternC Standards (c) (Bio-Rad).

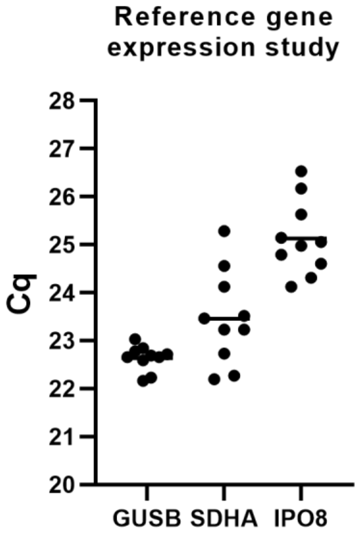

Supplementary Figure 3: Cq (quantification cycle) values of three reference genes, GUSB, SDHA (succinate dehydrogenase complex flavoprotein subunit A) and IPO8 (importin 8) in polarized ARPE-19 cells at 10 different time point within a period of 40 hours (0h, 4h, 8h, 12h, 16h, 20h, 28h, 32h, 36h and 40h). 7.5 ng cDNA was used for each PCR reaction. The values are represented in scatter dot plot format + mean (GUSB: mean±SD = 22.64±0.26; SDHA: mean±SD = 23.46±0.98; IPO8: mean±SD = 25.13±0.78).

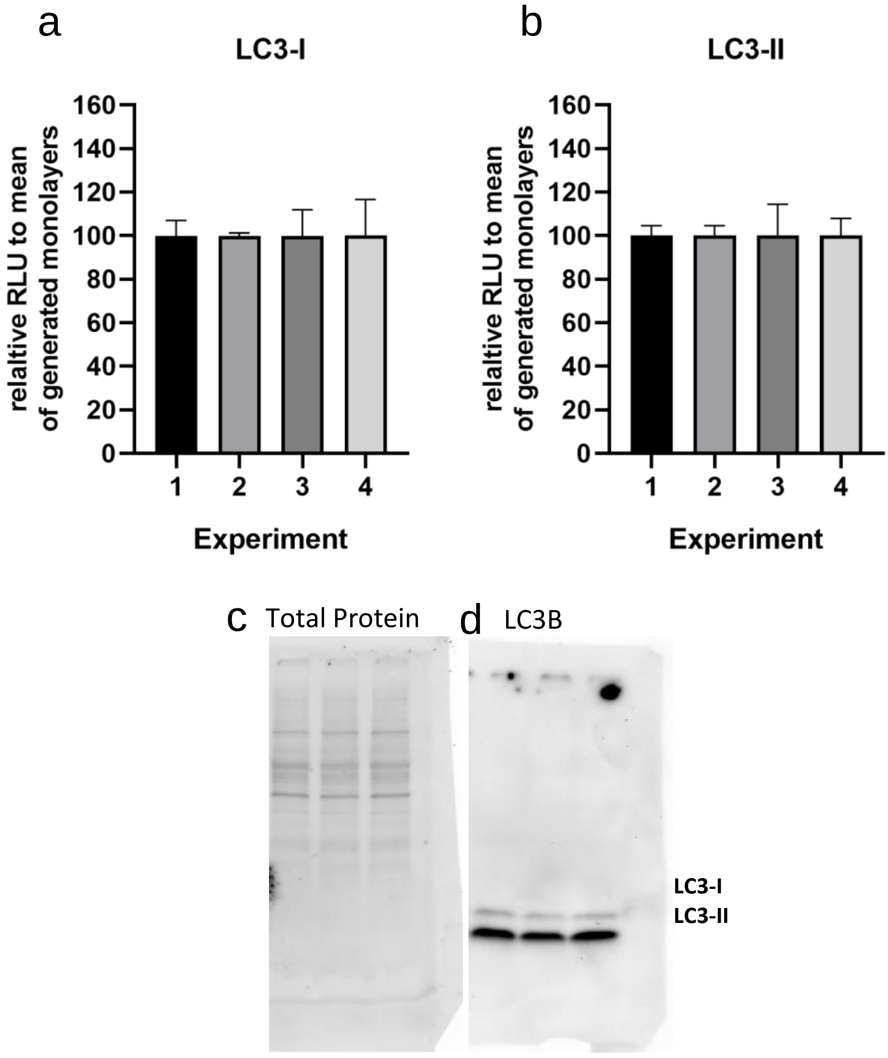

Supplementary Figure 4: LC3-I and LC3-II protein expression in polarized ARPE-19 cells. Relative a) LC3-I and b) LC3-II levels normalized to the amount of total loaded protein in generated monolayers. The cells of each experiment (n = 4) were thawed, expanded, seeded and differentiated at the same. The RLU levels LC3-I and LC3-II were relativized to the mean of generated monolayers of each experiment. Representative western blot analysis showing c) total protein loading and d) LC3-I and LC3-II in polarized ARPE-19 cells. The values are represented as mean±SD (n = 3-4).

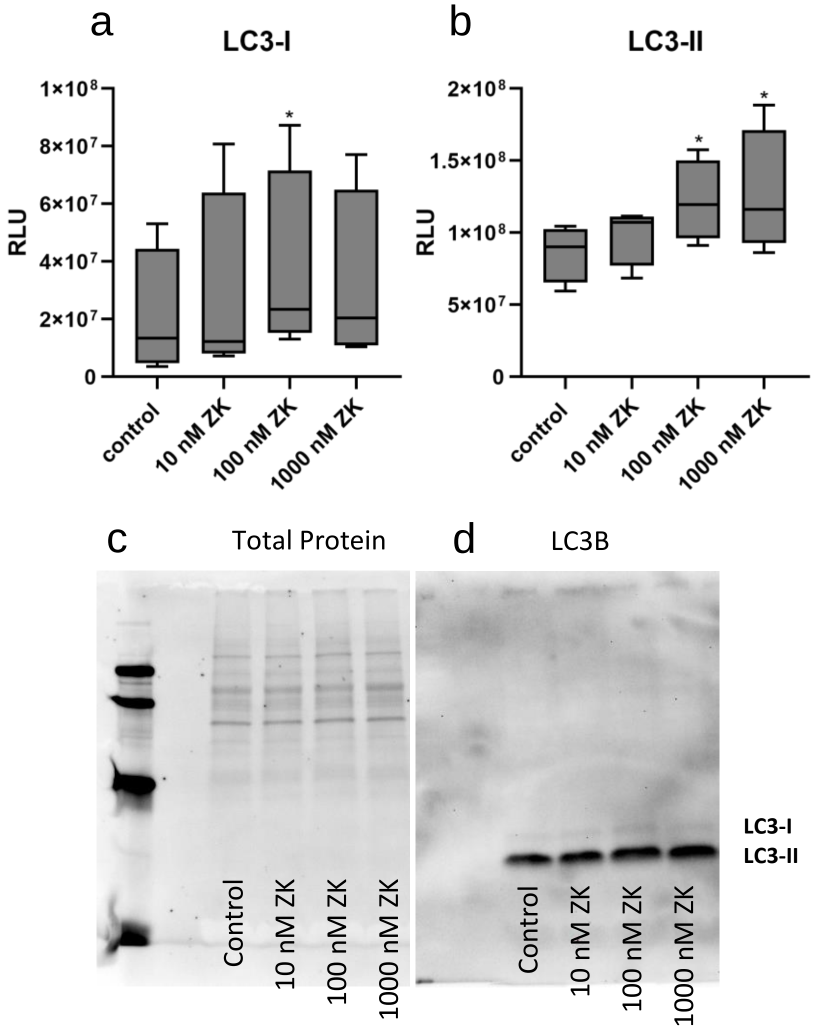

Supplementary Figure 5: LC3-I and LC3-II protein expression in polarized ARPE-19 cells treated with ZK. RLUs of a) LC3-I and b) LC3-II normalized to the amount of total loaded protein in polarized ARPE-19 cells treated with 10 nM, 100 nM and 1000 nM ZK for 3 hours. Representative western blot anlysis showing c) total protein loading and d) LC3-I and LC3-II in polarized ARPE-19 cells treated with ZK. ARPE-19 cells were cotreated with 10 µg/ml E64d and pepstatin A to prevent LC3-II degradation. The values are represented in box and whisker plot format (min to max); n = 4. Significance of LC3-I and LC3-II regulation upon ZK treatment was calculated by non-parametric repeated measures Friedman test followed by Dunn’s multiple comparison test. *p < 0.05.

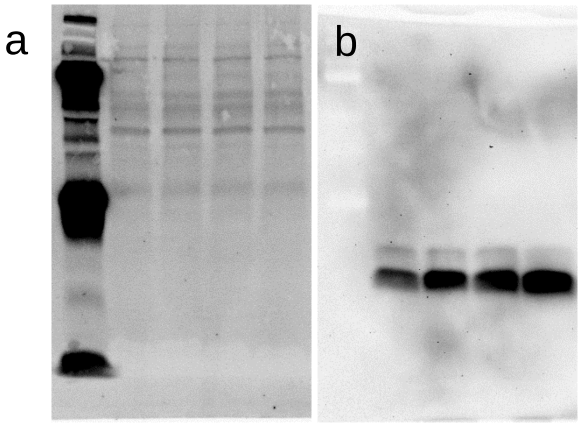

Supplementary Figure 6: Full length blot of figure 4. a) Total protein loading detected after blotting using stain-free imaging technology (Bio-Rad). b) Detection of LC3-I and LC3-II protein using recombinant Anti-LC3B antibody [EPR18709] (ab192890, Abcam). Marker: Precision Plus Protein WesternC Standards (Bio-Rad).

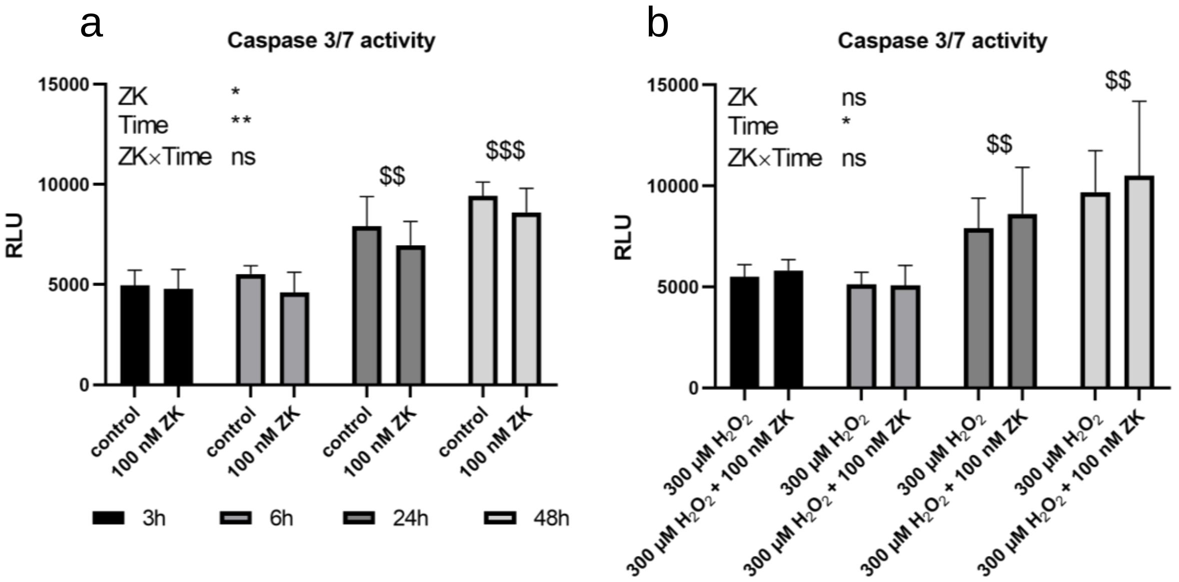

Supplementary Figure 7: Luminescence based caspase 3/7 activity assay on polarized ARPE-19 cells. Polarized ARPE-19 cells were treated with 100 nM ZK, 300 µM H_2O_2 or both 100 nM ZK + 300 µM H_2O_2 for 3, 6, 24 and 48 hours. Afterwards the cells were analyzed for caspase 3/7 activity under a) basal and b) oxidative stress conditions. The values are represented as mean±SD, n = 4. Significant impact on caspase 3/7 activity upon ZK, H_2O_2 and ZK×H_2O_2 treatment was calculated by repeated measures two-way ANOVA (main factors: ZK treatment (matched) and treatment time (matched); interaction: ZK×time) followed by Bonferroni's multiple comparison test. *p < 0.05, **p < 0.01; Main column effect: $$p < 0.01, $$$p < 0.001.

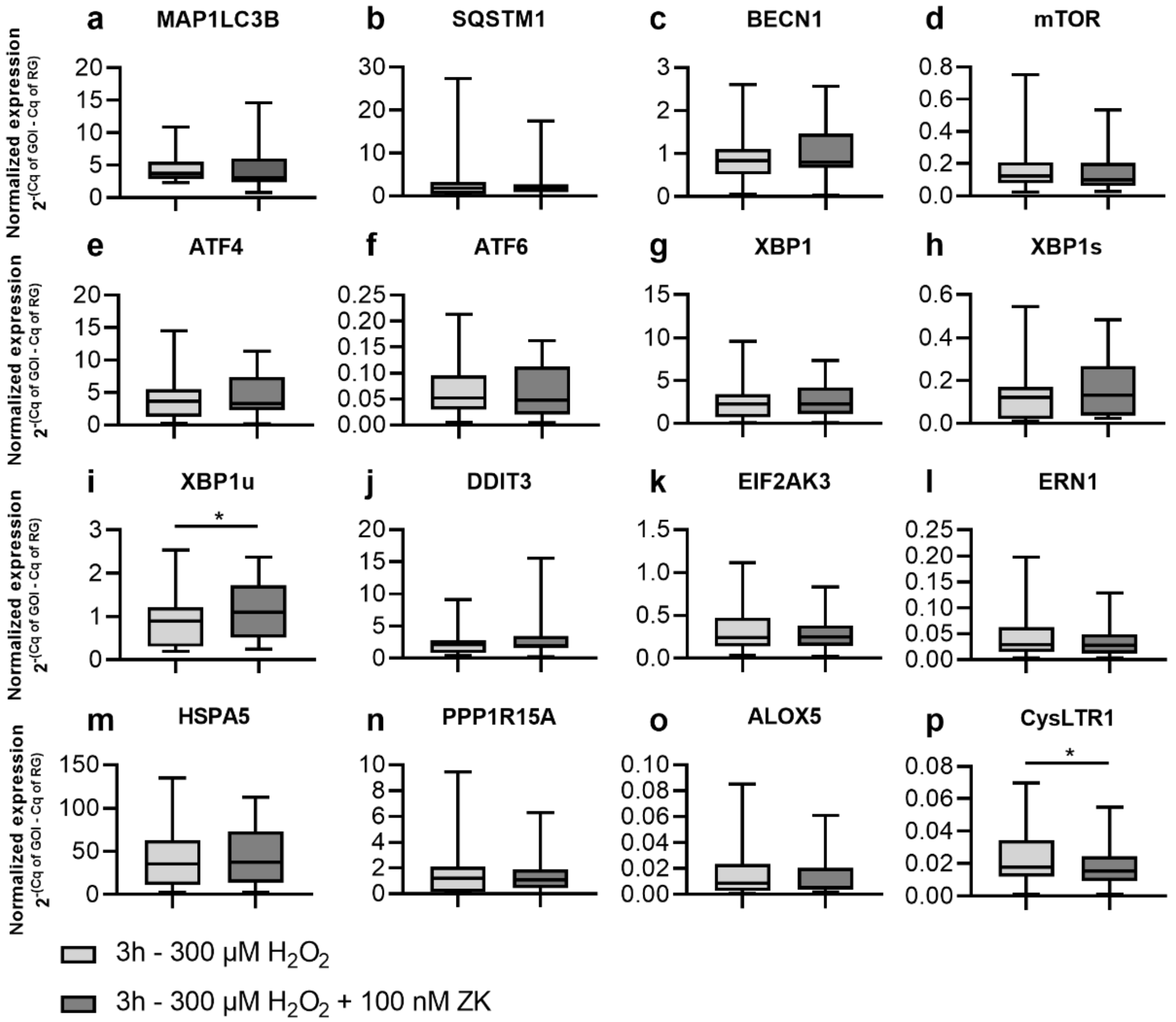

Supplementary Figure 8: Normalized expression of autophagosomal (a) MAP1LC3B, b) SQSTM1, c) BECN1 and d) mTOR), UPR-related (e) ATF4, f) ATF6, g) XBP1, h) XBP1s, i) XBP1u, j) DDIT3, k) EIF2AK3, l) ERN1, m) HSPA5, n) PPP1R15A), leukotriene synthesis (o) ALOX5, and p) CysLTR1) genes in polarized ARPE-19 cells, treated with 300 µM H_2O_2 alone or in combination with 100 nM ZK for 3 hours. The values are represented in box and whisker plot format (min to max); n = 15. Significance of gene regulation upon classification and ZK treatment was calculated with a paired t-test. *p < 0.05. Cq = quantification cycle; GOI = gene of interest; RG = reference gene.

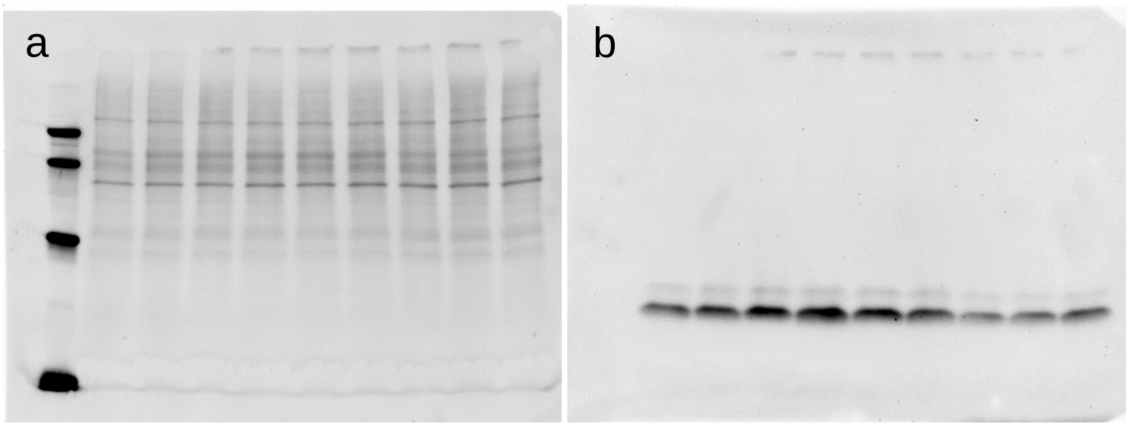

Supplementary Figure 9: Full length blot of figure 7. a, c) Total protein loading detected after blotting using stain-free imaging technology (Bio-Rad). b, d) Detection of LC3-I and LC3-II protein using recombinant Anti-LC3B antibody [EPR18709] (ab192890, Abcam). Marker: Precision Plus Protein WesternC Standards (Bio-Rad).
